# Body composition, balance, functional capacity and falls in older women

**DOI:** 10.1007/s40520-024-02719-5

**Published:** 2024-03-21

**Authors:** Yki Nordling, Reijo Sund, Joonas Sirola, Heikki Kröger, Masoud Isanejad, Toni Rikkonen

**Affiliations:** 1 Kuopio Musculoskeletal Research Unit, University of Eastern Finland, Kuopio, Finland; 2 Department of Orthopedics, Traumatology and Hand Surgery, Kuopio University Hospital, Kuopio, Finland; 3 Institute of Life Course and Medical Sciences, Department of Musculoskeletal and Ageing Sciences, University of Liverpool, 6 W Derby St, Liverpool, L7 8TX UK

**Keywords:** Body composition, Functional capacity, Balance, Falls, Injuries, Postmenopausal women

## Abstract

**Background:**

The aim of this study was to examine the association of body composition, muscle strength, balance, and functional capacity on falls and fall injuries among community-dwelling older women.

**Methods:**

The study comprised of a 2-year randomized controlled trial involving 914 women with an average age of 76.5 (SD = 3.3) years at baseline. The women were assigned to exercise intervention (*n* = 457) and control groups (*n* = 457). Clinical measurements were conducted at baseline, 12 months and 24 months.

**Results:**

During the 2-year follow up, total of 546 women (59.7%) sustained a fall. The total number of falls was 1380 and out of these, 550 (40%) of falls were non-injurious and 745 (54%) were injurious. Higher femoral neck bone mineral density (BMD) was associated with a higher overall risk of falls [RR = 2.55 (95% CI = 1.70–3.84, *p* < 0.001)], but was a protective factor for severe fall injuries [RR = 0.03 (95% CI = 0.003–0.035, *p* < 0.01)]. Slower Timed Up and Go (TUG) was associated with an increased overall risk of falls [RR = 1.07 (95% CI = 1.05–1.10, *p* < 0.001)] and injuries requiring medical attention [RR = 1.10 (95% CI = 1.02–1.19, *p* = 0.02)]. Longer single leg standing time was a protective factor for falls [RR = 0.99 (95% CI = 0.99–1.00, *p* < 0.01)] and overall injurious falls [RR = 0.99 (95% CI = 0.99–1.00, p = 0.02)].

**Conclusion:**

For postmenopausal women with higher femoral neck BMD appear to sustain more falls, but have a lower risk of severe fall injuries. Better TUG and single leg standing time predict lower risk of falls and fall injuries.

## Introduction

Fall injuries are a major cause of morbidity and mortality among older people and impose a significant economic and social burden [1]. Falls have been associated with deteriorating quality of life and an increasingly sedentary lifestyle [2]. Although several risk factors have been associated with falls, the magnitude of each of these risk factors in relation to the risk of falls has not been established [3].

Loss of muscle strength and functional capacity are among significant physiological changes among the older population [4]. Sarcopenia has been defined as the deterioration of muscle mass and decline in functional capacity [5–7] and is prevalent in the older population. Recent findings indicate that sarcopenia is negatively associated with bone mineral density (BMD) and increases the risk of hospitalization due to falls and fractures [8]. The evidence indicates that low BMD [4, 9–13] and sarcopenia [6, 14, 15] may be modifiable risk factors for falls and fractures in [6, 16]. Also, obesity has been proposed to increase the risk of falls in the population over 60 years of age [10]. Obesity is a common condition in older people that may have an effect on falls and risk of fracture. The ratio of body mass index (BMI) to the number of falls and hip fractures has been suggested to be non-linear [12, 17], but evidence pointing to a connection between obesity, fall-related injuries and fractures is sparse [18]. Physical exercise is shown to be an effective and safe way to maintain functional capacity while reducing osteoporosis, sarcopenia and related fall complications [15, 16, 19–21]. It has particularly been shown that regular strength and balance training can reduce the risk of falls in the elderly by 15–30% [22, 23] and fall-related fractures [16, 24].

The Kuopio fall prevention study (KFPS) was designed to study the health effects of exercise in community-dwelling older women and communal strategies to reduce falls and improve health. In this study, we investigated the relationship between initial muscle mass, body mass index, bone mineral density and functional capacity tests as a predictor of falls and fall injuries.

Methods.

### Study design and intervention

The Kuopio Fall Prevention Study (KFPS) is a 24-month randomized controlled trial, which was conducted to investigate the effects of an exercise intervention with fall prevention counseling on falls. The study protocol, power calculations [25] and main results [26] has previously been published in detail. The study was conducted by the Kuopio Musculoskeletal Research Unit (KMRU), University of Eastern Finland (UEF), in collaboration with the city of Kuopio, Finland, and involved 914 home-dwelling women (aged 72–84). The women were randomized into exercise intervention or control groups (457 + 457). The eligibility criteria for the study were: (1) women born between 1932 and 1945; (2) living within ≤ 10 km of Kuopio city center; (3) ability to attend the exercise sessions twice a week for the initial 6 months and (4) adequate health and independence (self-ambulatory, no unstable angina pectoris, severe pulmonary disease or moderate to severe dementia). All women who met the eligibility criteria were asked to take part in the two-year exercise trial. Within the study population, 582 women were recruited from the ongoing Kuopio Osteoporosis Risk Factor and Prevention (OSTPRE) Study—a population-based prospective cohort study. In addition, 332 women who were not included in the OSTPRE-cohort were recruited from the same central area. [25]

In the KFPS study, both groups received fall prevention counseling and lifestyle education twice during the first year. The intervention group participated in the supervised exercise intervention twice a week, including a one-hour gym training session and one Tai Chi class on separate days for the initial 6 months. In addition, the intervention group was provided with 12 months’ free use of all municipal sports facilities (including swimming pools, indoor halls, etc.) using a “Healthy-Kuopio” electronic keycard. Altogether, the study protocol was conducted from 2016 to 2019. The recruitment and baseline measurements for 914 women were completed in April 2017. The control group did not receive supervised exercise intervention or free access to communal facilities but was not restricted from participating in any activities at their own expense [25]. The mean participation rate for the supervised sessions was 70.4–79.6% coverage of the total sessions held depending on the group. The proportion of women attending over 80% of the supervised sessions was 61.9% (*n* = 283). On the contrary, 14 out of 457 women in the exercise group were non-compliant with 0 visits. The adherence was followed for the first 6 months. [26]

### Body composition and functional capacity measurements

Clinical measurements including muscle strength, functional capacity and mental state were performed by trained research personnel. Bone mineral density and total body soft tissue composition were measured using dual-energy X-ray absorptiometry (DXA) (Lunar iDXA, GE, Madison, Wisconsin, USA). DXA was used to measure fat mass and lean mass, as well as BMD in the total body and proximal femur (left hip). Measurements were repeated at baseline, 12 months, and 24 months [25].

Functional capacity, balance and muscle strength were measured with six different tests: (1) single leg standing, (2) squat, (3) isometric leg extension strength, (4) isometric handgrip strength (HGS) (Jamar, Sammons-Preston, Illinois, USA), (5) Timed Up and Go (TUG) and (6) postural sway analysis (HUR Labs BT3, Jyväskylä, Finland) [25].

One leg stand—test was performed using better foot and the best result of the two attempts was recorded. The maximum result was 30 s, and a result of less than 3 s was considered as a fail. In the squat test, the fingertips touched the floor. The test results were: (a) not able at all; (b) able to squat down completely and (c) able to get up from squat-down position without support (result: able/unable). Isometric strength tests was performed three times, and the best result was recorded. Dominant hand was used in the HGS-test. TUG-test measured the time (seconds) for a person rising from the chair, walking at normal speed to 3-m mark, turning, walking back to the chair and returning to the seated position. Postural sway analysis was performed standing on platform (a) normal stance, foot in V-position with eyes open, (b) normal stance, foot in V-position with eyes shut, (c) semitandem stance with eyes open and (d) semitandem stance with eyes shut.

### Physical activity

In the baseline questionnaire, the participants were asked about the frequency of physical activity that increases respiration rate or causes sweating. The question was “How often over the last year have you engaged in exercise that caused your respiratory rate to increased or caused you to sweat”: rarely, 1–3 times/month, 1 time/week, 2 times/week, 3 times/week, 4 times/week, 5 times/week or (almost) daily. Based on the responses, three groups were categorized: low physical activity (0–3 times physical activity per month) (*n* = 229), moderate physical activity (1–3 times per week) (*n* = 511), and high level of physical activity (more than three times per week) (*n* = 170).

### Falls and injuries

Information on falls and their prevailing circumstances was obtained by bi-weekly text messages (short message service, SMS) and positive falls reports were verified via a phone interview. All participants received a text message via cellphone twice a month asking whether they had fallen in the past 2 weeks. The text message was answered with a “yes” or “no”. If the subject answered yes, a phone interview was conducted to gain more information about the fall. The subject reported whether the outcome of the fall was: non-injurious, moderate injury or severe injury and whether the fall incident led to a doctor’s visit. Fractures due to falls were also confirmed in health registers.

The study diary included a fall survey that also recorded falls over 24 months for those women who could not participate in the text message follow-up (*n* = 108, 11.82%). In the same diary, the women also recorded additional physical activities, duration of exercise and any changes in health and medication.

The combined fall survey data were used in the statistical analysis to categorize the participants into the different groups according to the number of overall falls, non-injurious falls, moderate injury and severe injury and injurious falls requiring medical attention. The division of fall injuries into moderate and severe injuries was based on a fall questionnaire in which the person who fell answered whether the fall hurt moderately or severely according to her subjective feeling in terms of pain and functional constrain. The person also answered whether the fall led to a medical attention. For the statistical analysis, a category of any injurious fall was created by combining the subclasses of moderate and severe injury. Fractures were verified by perusal of medical records.

Almost half of all falls occurred on flat ground, either outdoors or indoors. The majority (68.9%) were caused by tripping or slipping. The study aimed to reduce bias due to seasonal factors (e.g., slipperiness in winter) by following all participants through the year(s). Fall characteristics, including fall mechanism and terrain, have been reported previously in detail. [26]

### Statistical methods

An independent samples T-test was used to compare means (SD) of continuous variables and a chi-square test for categorical variables *n* (%). Baseline measurements of body composition, functional capacity and muscle strength were compared between those women who fell at least once during the follow-up and those who did not. For the statistical analysis of body composition, the following variables were constructed: body mass index (BMI) = weight/height^2^ (kg/m^2^), appendicular lean mass (ALM) = lean mass of arms + lean mass of legs (kg), ALM/height^2^ (ALM/ht^2^, kg/m^2^) and ALM/BMI (m^2^). ALM/ht^2^ and ALM/BMI have been used to represent lean mass standardized to body size [27].

A Poisson regression analysis was used to examine the association between independent variables and the frequency of subclasses of falls. Functional tests, strength and body composition variables were analyzed using age and treatment group adjusted univariate model and certain variables were selected for the multivariate model. In the age and treatment-adjusted univariate Poisson regression model independent variables were BMD, fat and lean mass from the DXA analysis, BMI, ALM, ALM/ht^2^, ALM/BMI, balance, functional capacity and muscle strength measurements. Age, treatment group (intervention/control), fBMD, BMI, HGS and TUG were selected for the multivariate model based on the results of the univariate model and the general usability of the variables. fBMD, BMI and TUG are widely used variables in medical evaluation. The offset (time) variable included the follow-up duration, from the baseline to last confirmed fall information, including the latest text message response date, returned fall diary, or end of the 24-month follow-up, whichever occurred first. Analyses were conducted using the SPSS statistical software program (IBM Corp. Released 2020. IBM SPSS Statistics for Windows. Version 27.0. Armonk, NY: IBM Corp).

## Results

The mean baseline age of the women was 76.5 years (SD = 3.3). During the 2-year follow up (mean follow up time 1.88 years, SD = 0.40), 546 women (59.7%) fell at least once. The total number of reported falls was 1380, whereas the individual number of falls during the follow-up ranged from 0 to 28. Out of these, 550 (40%) of falls were non-injurious and 745 (54%) were injurious with moderate (*n* = 681) or severe (*n* = 61) fall injury. Out of these, 171 (12%) of falls required medical attention, with 63 women having one or more fractures. For 85 of the falls (6%), detailed fall data (phone interview) were not available. In the comparison between fallers and non-fallers, the baseline value of the total body bone mass was 1.9% higher (*p* = 0.06) and bone mass of the legs was 2.6% higher (*p* = 0.01) in fallers. Also, the BMD of the femoral neck was 2.4% higher (*p* = 0.03) in the group who fell at least once. There was no difference in baseline measurements of functional capacity or muscle strength measurements between fallers and non-fallers during the trial (Table 1).

**Table 1:** Main characteristic comparison between fallers and non-fallers during follow-up

Baseline Characteristics	Mean (Sd)	Non-Fallers (*N* = 367)	Fallers (*N* = 546)	*p* values
Age, years	76.5 (3.2)	76.8 (3.4)	76.4 (3.2)	0.09
Height, cm	159 (5.5)	158 (5.4)	159 (7.2)	0.02
Weight, kg	68.4 (12.1)	68.7 (12.1)	69.4 (12.4)	0.51
Waistline, cm	90.9 (11.7)	90.7 (11.9)	91.1 (11.6)	0.63
Body mass index, kg/m^2^	27.3 (4.5)	27.4 (4.7)	27.2 (4.4)	0.66
Body composition measurements (DXA), g				
Leg fat mass	8869 (2858)	8826 (2998)	8894 (2764)	0.92
Leg lean mass	12,995 (1821)	12,956 (1824)	13,019 (1821)	0.54
Leg bone mass	759 (113)	747 (106)	767 (117)	0.01
Total fat mass	27,728 (8639)	27,667 (8984)	27,760 (8413)	0.87
Total lean mass	38,942 (4249)	38,761 (4116)	39,058 (4337)	0.30
Total bone mass	2075 (303)	2053 (294)	2091 (208)	0.06
Femoral neck BMD, g/cm^2^	0.843 (0.127)	0.83 (0.12)	0.85 (0.13)	0.03
Body composition derivates				
Appendicular lean mass (kg)	17.08 (2.34)	17.04 (2.34)	17.11 (2.37)	0.66
ALM/height^2^ (kg/m^2^)	6.80 (1.67)	6.78 (0.85)	6.81 (2.04)	0.79
ALM/BMI (m^2^)	0.63 (0.08)	0.63 (0.08)	0.64 (0.08)	0.40
Functional capacity				
Timed Up and Go, seconds	9.7 (1.9)	9.87 (2.06)	9.69 (1.88)	0.18
Balance				
Single leg standing, seconds	15.3 (11.3)	14.61 (11.25)	15.84 (11.33)	0.11
Sway analyses				
Feet in V- position with eyes open, mm	181 (116)	174 (115)	185 (116)	0.14
Feet in V- position with eyes closed, mm	281 (213)	273 (217)	286 (211)	0.40
Semitandem stance with eyes open mm	262 (154)	254 (134)	267 (166)	0.37
Semitandem stance with eyes closed, mm	584 (469)	532 (316)	618 (545)	0.38
Muscle strength				
Knee extension, Nm	290 (69)	290 (72)	291 (67)	0.30
Hand grip strength, kg	26 (5)	26 (5.3)	26 (4.8)	0.69

An independent samples T-test was used to compare the means (SD) of the baseline characteristics between the fallers and non-fallers

Appendicular lean mass (ALM) = lean mass arms + lean mass legs

### Association between body composition with falls

The results of the 2-year follow-up indicated that after adjusting for age and treatment group, a one-unit higher BMI was associated with a lower risk of non-injurious falls (RR = 0.97, 95% CI = 0.95–0.99, *p* < 0.01) (Table 2). In the DXA measurements (Table 2), a one-unit higher femoral neck BMD (g/cm^2^) increased the overall risk of falls (RR = 2.55, 95% CI = 1.70–3.84, *p* < 0.001), the risk of non-injurious falls (RR = 2.45, 95% CI = 1.28–4.67, p = 0.01) and the risk of injurious falls (RR = 2.02, 95% CI = 1.15–3.53, *p* = 0.01). A higher femoral neck BMD was associated with an increased risk of moderate fall injuries (RR = 2.71, 95% CI = 1.52–4.82, *p* < 0.01) but a lower risk of severe fall injuries (RR = 0.03, 95% CI = 0.003–0.35, *p* < 0.01) and fractures (RR = 0.03, 95% CI = 0.002–0.30, *p* < 0.01).

**Table 2:** Association between BMI and body composition and the subclasses of falls. (RR, 95% CI)

Variable	Overall falls (*n* = 1380)	Non-injurious falls *n* = 550)	Overall injurious falls (Moderate + Severe injuries, (*n* = 745)	Subclass [Moderate injuries (*n* = 684)]	Subclass [Severe injuries *n* = 61)]	Injurious falls requiring medical attention (*n* = 171)	Fractures (*n* = 63)
BMI	0.99 (CI = 0.98–1.00, *p* = 0.12)	0.97 (CI = 0.95–0.99, *p* < 0.01)	1.00 (CI = 0.98–1.01, *p* = 0.65)	1.00 (CI = 0.98–1.02, *p* = 0.89)	0.97 (CI = 0.91–1.03, *p* = 0.25)	1.00 (CI = 0.96–1.03, *p* = 0.79)	0.96 (CI = 0.90–1.01, *p* = 0.14)
ALM	1.01 (CI = 0.99–1.04, *p* = 0.31)	1.00 (CI = 0.96–1.04, *p* = 0.96)	1.02 (CI = 0.99–1.05, *p* = 0.20)	1.02 (CI = 0.99–1.06, *p* = 0.16)	0.99 (CI = 0.88–1.10, *p* = 0.80)	1.01 (CI = 0.94–1.07, *p* = 0.88)	1.00 (CI = 0.90–1.12, *p* = 0.96)
ALM/height^2^	0.84 (CI = 0.96–1.03, *p* = 0.84)	1.01 (CI = 0.96–1.06, *p* = 0.68)	0.97 (CI = 0.90–1.04, *p* = 0.33)	0.97 (CI = 0.90–1.04, *p* = 0.38)	0.93 (CI = 0.70–1.26, *p* = 0.65)	0.96 (CI = 0.82–1.12, *p* = 0.58)	0.91 (CI = 0.67–1.24, *p* = 0.56)
ALM/BMI	3.25 (CI = 1.69–6.25, *p* < 0.001)	8.29 (CI = 3.01–22.83, *p* < 0.001)	3.29 (CI = 1.35–8.01, *p* = 0.01)	2.96 (CI = 1.17–7.49, *p* = 0.02)	10.51 (CI = 0.50–219.55, *p* = 0.13)	2.07 (CI = 0.32–13.39, *p* = 0.44)	29.47 (CI = 1.59–547.87, *p* = 0.02)
Bone mass (legs)	1.00 (CI = 1.00–1.00, *p* < 0.001)	1.00 (CI = 1.00–1.00, *p* < 0.001)	1.00 (CI = 1.00–1.00, *p* < 0.001)	1.00 (CI = 1.00–1.00, *p* < 0.001)	1.00 (CI = 0.99–1.00, *p* = 0.01)	1.00 (CI = 1.00–1.00, p = 0.51)	1.00 (CI = 0.99–1.00, *p* = 0.02)
Bone mass (trunk)	1.00 (CI = 1.00–1.00, *p* < 0.01)	1.00 (CI = 1.00–1.00, *p* = 0.15)	1.00 (CI = 1.00–1.00, *p* = 0.07)	1.00 (CI = 1.00–1.00, *p* = 0.01)	1.00 (CI = 1.00–1.00, *p* = 0.004)	1.00 (CI = 1.00–1.00, *p* = 0.02)	1.00 (CI = 1.00–1.00, *p* = 0.012)
Bone mass (total)	1.00 (CI = 1.00–1.00, *p* < 0.001)	1.01 (CI = 1.00–1.02, *p* < 0.001)	1.00 (CI = 1.00–1.00, *p* < 0.001)	1.00 (CI = 1.00–1.00, *p* < 0.001)	1.00 (CI = 1.00–1.00, *p* < 0.001)	1.00 (CI = 1.00–1.00, *p* = 0.11)	1.00 (CI = 1.00–1.00, *p* < 0.004)
Bone mass density							
Femoral neck	2.55 (CI = 1.70–3.84, *p* < 0.001)	2.45 (CI = 1.28–4.67, *p* = 0.01)	2.02 (CI = 1.15–3.53, *p* = 0.01)	2.71 (CI = 1.52–4.82, *p* = 0.001)	0.03 (CI = 0.003–0.35, *p* = 0.004)	0.38 (CI = 0.11–1.36, *p* = 0.14)	0.03 (CI = 0.002–0.30, *p* < 0.003)
Trochanter	2.24 (CI = 1.49–3.38, *p* < 0.001)	1.62 (CI = 0.85–3.11, *p* = 0.14)	1.87 (CI = 1.07–3.27, *p* = 0.03)	2.69 (CI = 1.51–4.79, *p* = 0.001)	0.02 (CI = 0.00–0.16, *p* < 0.001)	0.20 (CI = 0.06–0.70, *p* = 0.01)	0.82 (CI = 0.72–0.92, *p* < 0.002)

The analysis was conducted using Poisson regression adjusted for age and treatment group

*RR* Risk Ratio, *CI* Confidence Interval, *p*-value

Appendicular lean mass (ALM) lean mass arms + lean mass legs

All variables are continuous

According to the DXA data, lean mass was not directly associated with falls or fall injuries, but higher ALM/BMI increased the risk of falls (RR = 3.25, 95% CI = 1.69–6.25, *p* < 0.001), non-injurious falls (RR = 8.29, 95% CI = 3.01–22.83, *p* < 0.001) and injurious falls (RR = 3.29, 95% CI = 1.35–8.01, *p* = 0.01). A higher ALM/BMI increased the risk of moderate injuries (RR = 2.96, 95% CI = 1.17–7.49, *p* = 0.02), but there was no association between ALM/BMI and severe fall injuries or injuries requiring medical attention. No statistically significant association was found between ALM or ALM/ht^2^ and falls.

### Association between functional capacity, balance and muscle strength with falls

In the Poisson regression analysis, adjusted for age and treatment group allocation, a longer time in the TUG test (s) was statistically significantly in relation to overall risk of falls (RR = 1.07, 95% CI = 1.05–1.10, *p* < 0.001), the overall risk of injurious falls (RR = 1.06, 95% CI = 1.02–1.10, *p* = 0.001), the risk of injurious falls requiring medical attention (RR = 1.10, 95% CI = 1.02–1.18, *p* = 0.01) and fracture risk (RR = 1.21, 95% CI = 1.01–1.33, *p* < 0.01) (Table 3).

**Table 3:** Association between balance, mobility and strength and the subclasses of falls. (RR, 95% CI)

Variable	Overall falls (*n* = 1380)	Non-injurious falls *n* = 550)	Overall injurious falls (Moderate + Severe injuries, (n = 745)	Subclass [Moderate injuries (*n* = 684)]	Subclass [Severe injuries *n* = 61)]	Injurious falls requiring medical attention (*n* = 171)	Fractures (*n* = 63)
Balance							
single leg standing	0.99 (CI = 0.99–1.00, *p* = 0.001)	1.00 (CI = 1.00–1.01, *p* = 0.33)	0.99 (CI = 0.99–1.00, *p* = 0.02)	0.99 (CI = 0.98–1.00, *p* = 0.001)	1.00 (CI = 0.98–1.03, *p* = 0.81)	1.00 (CI = 0.99–1.01, *p* = 0.94)	1.00 (CI = 0.97–1.02, *p* = 0.74)
Functional capacity							
Timed Up and Go	1.07 (CI = 1.05–1.10, *p* < 0.001)	1.00 (CI = 0.96–1.05, *p* = 0.96)	1.06 (CI = 1.02–1.10, *p* < 0.002)	1.07 (CI = 1.03–1.11, *p* < 0.001)	0.99 (CI = 0.87–1.14, *p* = 0.93)	1.10 (CI = 1.02–1.18, *p* = 0.01)	1.21 (CI = 1.01–1.33, *p* < 0.001)
Strength							
Knee extension (left)	1.001 (CI = 1.000–1.002, *p* = 0.002)	1.003 (CI = 1.002–1.005, *p* > 0.001)	1.001 (CI = 1.000–1.002, *p* = 0.145)	1.00 (CI = 1.00–1.00, *p* = 0.10)	0.999 (CI = 0.995–1.003–1.00, *p* = 0.591)	0.998 (CI = 0.996–1.000, *p* = 0.046)	0.997 (CI = 0.993–1.001, *p* = 0.111)
Knee extension (right)	1.000 (CI = 1.000–1.001, *p* = 0.24)	1.002 (CI = 1.001–1.003, *p* = 0.001)	1.00 (CI = 1.00–1.00, *p* = 0.96)	1.000 (CI = 0.999–1.001, *p* = 0.961)	0.999 (CI = 0.996–1.003, *p* = 0.710)	0.998 (CI = 0.996–1.000, *p* = 0.082)	0.998 (CI = 0.995–1.002, *p* = 0.356)
HGS	1.00 (CI = 0.99–1.01, *p* = 0.64)	1.01 (CI = 1.00–1.03, *p *= 0.12)	1.01 (CI = 0.99–1.02, *p* = 0.99)	1.00 (CI = 0.98–1.01, *p* = 0.84)	1.02 (CI = 0.97–1.07, *p* = 0.50)	0.98 (CI = 0.95–1.01, *p* = 0.10)	1.00 (CI = 0.95–1.06, *p* = 0.91)

Age and treatment group adjusted for balance, functional capacity and strength measurements. The moderate and severe fall injuries classes are subclasses of the total injurious falls class

The analysis was conducted using Poisson regression adjusted for age and treatment group

*RR* Risk Ratio, *CI* Confidence Interval, *p*-value, H*GS* hand grip strength

All variables are continuous

Balance was measured as a single leg standing test and computerized postural sway analysis. A longer single leg standing time was statistically significantly associated with a lower overall risk of falls (RR 0.99, 95% CI = 0.99–1.00, *p* < 0.01) and overall injurious falls (RR = 0.99, 95% CI = 0.99–1.00, *p* = 0.02). Postural sway was not associated with falls or fall injuries.

In this study, the results suggest that better knee extension strength (Table 3) seems to slightly increase the risk of falls and the risk of non-injurious falls. On the other hand, better leg extension strength also seems to provide some protection against falls requiring a medical attention. Isometric extension of the left knee was statistically significantly associated with higher risk of total number of falls (RR = 1.001, CI = 1.000–1.002, *p* = 0.002). Left leg (RR = 1.003, CI = 1.002–1.005, *p* > 0.001) and right leg (RR 1.002 (CI = 1.001–1.003, *p* = 0.001) isometric knee extension strength increased the risk of non-injurious falls. Risk of injurious falls requiring medical attention was lower with better knee extension strength [left leg: RR 0.998 (CI = 0.996–1.000, *p* = 0.046) and right leg RR 0.998 (CI = 0.996–1.000, *p* = 0.082).Association between physical activity and falls.

The association between physical activity and fall and injury risk was compared to high physical activity in the age- and treatment-group adjusted Poisson regression analysis. The results are shown in Table 4. Low physical activity (RR 0.80, 95% CI = 0.63–1.03, *p* = 0.08) and moderate physical activity (RR = 0.50, 95% CI = 0.66–1.00, *p* = 0.05) may reduce the risk of non-injurious falls compared to high physical activity. In terms of a severe fall injury, low self-reported physical activity (RR = 2.58, 95% CI = 0.95–7.00, *p* = 0.06) and moderate self-reported physical activity (RR = 2.61, 95% CI = 1.03–6.62, *p* = 0.04) were risk factors compared to high physical activity. Low physical activity (RR = 1.57, 95% CI = 0.98–2.53, *p* = 0.06) also indicated a risk of injurious falls requiring medical attention compared to high physical activity.

**Table 4:** Association between physical activity and the subclasses of falls. (RR, 95% CI)

Variable	Overall falls (*n* = 1380)	Non-injurious falls *n* = 550)	Overall injurious falls (Moderate + Severe injuries, (*n* = 745)	Subclass [Moderate injuries (*n* = 684)]	Subclass [Severe injuries *n* = 61)]	Injurious falls requiring medical attention (*n* = 171)	Fractures (*n* = 63)
Physical activity that causes sweating and increased respiratory rate							
Low physical activity [0–3 times per month (*n* = 229)]	0.99 (CI = 0.84–1.15, *p* = 0.86)	0.80 (CI = 0.63–1.03, *p* = 0.08)	0.99 (CI = 0.79–1.23, *p* = 0.90)	0.93 (CI = 0.74–1.16, *p* = 0.52)	2.58 (CI = 0.95–7.00, *p* = 0.06)	1.57 (CI = 0.98–2.53, *p* = 0.06)	1.63 (CI = 0.66–4.00, *p* = 0.29)
Moderate physical activity [1–3 times per week (*n* = 511)]	0.90 (CI = 0.78–1.03, *p* = 0.12)	0.50 (CI = 0.66–1.00, *p* = 0.05)	0.72 (CI = 0.80–1.17, *p* = 0.97)	0.91 (CI = 0.74–1.10, *p* = 0.32)	2.61 (CI = 1.03–6.62, *p* = 0.04)	1.25 (CI = 0.81–1.95, *p* = 0.32)	1.96 (CI = 0.88–4.38, *p* = 0.10)
High physical activity [Over 3 times per week (*n* = 170)]	1	1	1	1	1	1	1

Age and treatment group adjusted for physical activity parameters. Poisson regression analysis was used

*RR* risk Ratio, *CI* Confidence Interval, *p* p-value

The intermediate and severe fall injuries classes are subclasses of the overall injurious falls class

Based on the frequency of physical activity that caused increased respiratory rate and sweating, the women were divided into three groups. Information on physical activity was obtained from the baseline questionnaire

Lower levels of physical activity have been compared with higher levels of physical activity

### Fall and injury risk

All multivariable models related to falls and fall injuries were adjusted for age, intervention group, femoral neck BMD, BMI, HGS, and TUG. The results of the multivariable model are shown in Table 5. In the multivariable model, a higher risk of falls was associated with femoral BMD (RR = 3.03, 95% CI = 1.99–4.62, *p* < 0.001) and longer TUG time (RR = 1.10, 95% CI = 1.07–1.13, *p* < 0.001). A lower risk of falls was associated with higher age (RR = 0.96, 95% CI = 0.95–0.98, *p* < 0.001) and BMI (RR = 0.97, 95% CI = 0.096–0.99, *p* < 0.001). A higher BMI was also associated with a lower risk of non-injurious falls (RR = 0.97, 95% CI = 0.96–0.99, *p* < 0.001) but had no association with fall injuries. Also, a higher femoral BMD (RR = 3.42, 95% CI = 1.76–6.66, *p* < 0.001) and stronger HGS (RR = 1.02, 95% CI = 1.00–1.04, *p* = 0.06) increased the risk of non-injurious falls.

**Table 5:** Association between age, treatment group, BMD, BMI, HGS, TUG and the subclasses of falls in the multivariable model. (RR, 95% CI)

Variable	Overall falls (*n* = 1380)	Non-injurious falls *n* = 550)	Overall injurious falls (Moderate + Severe injuries, (*n* = 745)	Subclass [Moderate (*n* = 684)]	Subclass [Severe injuries *n* = 61)]	Injurious falls requiring medical attention (*n* = 171)	Fractures (*n* = 63)
Age	0.96 (CI = 0.95–0.98, *p* < 0.001)	0.94 (CI = 0.92–0.97, *p* < 0.001)	0.95 (CI = 0.93–0.98, *p* < 0.001)	0.95 (CI = 0.92–0.97, *p* < 0.001)	1.02 (CI = 0.94–1.11, *p* = 0.66)	0.93 (CI = 0.88–0.98, *p* = 0.01)	1.01 (CI = 0.93–1.09, *p* = 0.84)
Treatment (intervention group and control group)	1.16 (CI = 1.05–1.29, *p* = 0.01)	1.01 (CI = 0.92–1.29, *p* = 0.33)	1.06 (CI = 0.91–1.22, *p* = 0.46)	1.03 (CI = 0.89–1.20, *p* = 0.70)	1.43 (CI = 0.85–2.42, *p* = 0.18)	1.18 (CI = 0.86–1.60, *p* = 0.30)	1.59 (CI = 0.95–2.66, *p* = 0.08)
Femoral neck BMD	3.03 (CI = 1.99–4.62, *p* < 0.001)	3.42 (CI = 1.76–6.66, *p* < 0.001)	2.22 (CI = 1.24–3.97, *p* = 0.01)	2.94 (CI = 1.63–5.34, *p* < 0.001)	0.04 (CI = 0.00–0.47, *p* = 0.18)	0.41 (CI = 0.11–1.53, *p* = 0.18)	0.04 (CI = 0.003–0.450, *p* = 0.01)
BMI	0.97 (CI = 0.96–0.99, *p* < 0.001)	0.96 (CI = 0.93–0.98, *p* < 0.001)	0.98 (CI = 0.97–1.00, p = 0.07)	0.98 (CI = 0.97–1.00, *p* = 0.08)	0.98 (CI = 0.92–1.05, *p* = 0.55)	0.99 (CI = 0.96–1.03, *p* = 0.74)	0.96 (CI = 0.90–1.02, *p* = 0.15)
HGS	1.01 (CI = 1.00–1.01, *p* = 0.13)	1.02 (CI = 1.00–1.04, *p* = 0.06)	1.00 (CI = 0.99–1.02, p = 0.67)	1.00 (CI = 0.99–1.02, *p* < 0.79)	1.02 (CI = 0.90–1.19, *p* = 0.50)	0.98 (CI = 0.95–1.01, *p* = 0.23)	1.03 (CI = 0.98–1.08, *p* = 0.31)
TUG	1.10 (CI = 1.07–1.13, *p* < 0.001)	1.04 (CI = 0.99–1.09, *p* = 0.11	1.07 (CI = 1.03–1.12, *p* < 0.001)	1.08 (CI = 1.03–1.12, *p* < 0.001)	1.04 (CI = 0.90–1.19, *p* = 0.63)	1.10 (CI = 1.02–1.19, *p* = 0.02)	1.25 (CI = 1.12–1.34, *p* < 0.001)

Multivariable Poisson analysis of age, intervention group, femoral neck BMD, BMI, HGS and TUG

*RR* Risk Ratio, *CI* Confidence Interval, *p* p-value

Risk of falls, non-injurious falls and injurious falls. The moderate and severe fall injuries classes are subclasses of the total injurious falls class

*BMD* bone mineral density, *BMI* body mass index

*HGS* hand grip strength; *TUG* timed Up and Go

All variables, except the treatment variable, are continuous

The treatment variable is dichotomous (intervention group and control group)

A slower TUG result increased the risk of injurious falls requiring medical attention (RR = 1.10, 95% CI = 1.02–1.19, *p* = 0.02) and fractures (RR = 1.25, 95% CI = 1.12–1.34, *p* < 0.001). A higher femoral BMD was associated with reduced fracture risk (RR = 0.04, 95% CI = 0.00–0.45, *p* = 0.01). Higher age reduced the risk of moderate injuries (RR = 0.95, 95% CI = 0.92–0.97, *p* < 0.001) and injurious falls requiring medical attention (RR = 0.93, 95% CI = 0.88–0.98, *p* = 0.01).

## Discussion

The findings of this study indicated that a higher femoral BMD results in a higher fall frequency although it is a protective factor for severe fall injuries. A faster performance in the TUG test and a longer performance in the one-leg standing test were protective factors against falls and fall-related injuries. A higher ALM/BMI was associated with overall risk of falls, including non-injurious falls and moderate fall injuries, but not with severe fall injuries. The results suggest that physically active older women with favorable functional capacity, balance and body composition sustain a higher number of falls. However, they have protective factors against severe fall injuries such as fractures.

Better functional capacity and balance have previously been found to protect against fall injuries [4], which is aligned with the results of this study. For the TUG test, a one second faster performance increment reduced the risk of overall falls by 7%, overall injurious falls by 6% and fall injuries requiring medical attention by 9%. A 1 s increment in single-leg standing time reduced the overall risk of falls and the overall risk of injurious falls by 1%. These results are in alignment with previous findings in which a one second longer single leg standing time in older women was associated with a reduced risk of hip fracture by 5% [27]. Also, in a previous study [28], simple functional tests such as single leg standing for 10 s, low HGS and inability to squat showed significant prediction for hip fracture risk in aging women The results of this study suggest that the prevention of fall injuries should aim to improve or maintain functional capacity and balance.

In addition to functional capacity and balance, this study investigated the association between muscle strength and muscle mass with falls and fall-related injuries. No direct association was found between muscle mass and falls or injuries. A higher ALM/BMI was associated with a higher risk of falls and moderate fall injuries, but not with severe fall injuries. Of the muscle strength variables, a stronger HGS was associated with the risk of non-injurious falls. Better knee extension muscle strength was statistically significantly associated with higher risk of non-injurious falls but lower risk of injurious falls requiring medical attention. The findings suggest that people with better muscle strength are more likely to sustain a fall more frequently, although the risk of injury is not increased. Among the older Australian population, men and women whose grip strength was below the 25th percentile had a higher risk of falling over 1 year. A similar finding was reported in the Swedish population, where grip strength was a predictor of incident falls [29]. However, no injurious falls were reported [30]. According to previous research [31], HGS appears to be a predictor of mobility, balance and activities of daily living. However, more studies are warranted to investigate whether HGS can predict injurious falls.

There was also no significant association between obesity and self-reported fall injuries. A higher BMI was associated with a lower risk of non-injurious falls. The results may be due to the homogenous study population since the mean value of BMI in this study was 27 kg/m^2^ (SD 4.5), which is associated with the lowest mortality rate in older adults [32] and lower hip fracture risk [12] compared to lower and higher BMI. According to the meta-analysis by Neri et al. [18], obesity has been suggested to increase the overall fall risk in people aged 60 years and older. Evidence of the association between obesity and fall-related injuries or fractures is insufficient. Obesity challenges balance control due to altered postural strategy, which can be seen in the higher center of pressure speed [33]. According to a recent meta-analysis [34] center of pressure displacement characteristics differentiate fall risk in older people and is suggested to be useful for follow-up in postural balance. In this study, sway analysis was not associated with falls.

The DXA data showed that higher femoral neck BMD was associated with an increased overall risk of falls and non-injurious falls. However, it was a protective factor for severe fall injuries. The findings from previous studies show that an increase in total hip BMD has been associated with decreased major osteoporotic fractures compared with stable BMD, whereas lower BMD and a decrease in BMD have been associated with a higher risk of fractures [28, 35]. In general, the current data suggest that higher femoral BMD is associated with a lower risk of severe fall injuries, such as fractures in older women.

The different association of BMD with risk of falls and risk of injurious falls is likely explained by the physical activity. Physical activity improves physical capacity and BMD [11, 16, 19, 21], which in turn may protect older people from serious fall injuries such as fractures [13, 24]. However, physical activity also increases the overall exposure to factors such as outdoor elements, obstacles, and terrain, which elevate the risk of fall [3].

In this study, physical activity was not associated with overall fall risk. However, inactivity seems to increase the risk of severe fall injuries compared to women who engage in higher level of physical activity. Minimizing sedentary time is important since it has a positive association with a higher risk of fractures [36] and overall mortality [37, 38]. To prevent fall injuries, older people may benefit from services that promote physical activity [36]. The results of the recent large-scale RCT suggest that light- or moderate-intensity exercise group programs can reduce falls and fall injuries among older women on a municipal scale [26].

The results of this study suggest that good functional capacity and bone density protect against fall-related injury. Many factors such as higher ALM/BMI, HGS or physical activity increased the risk of falls but were not associated with fall injuries. In the future, fall prevention research on community-dwelling older women should focus more on fall injuries instead of overall fall incidence as an outcome.

Strengths and limitations.

The strengths of this study are the large sample size, long follow-up time, extensive fall record, and clinical measurement protocol. This study particularly addresses the importance of fall data representation to reflect the number of falls and whether or not they are injurious. The high number of falls, with non-injurious falls and falls requiring medical attention, allowed for a conclusive statistical analysis to be performed. The majority of falls were recorded from biweekly text message questions with yes/no answers, which most likely reduced the risk of recalling errors. Phone interviews within 2 weeks of an incident ensured that the most accurate and relevant information about the falls was obtained.

This study also has some limitations. The level of injury classification was based on the level of subjective pain (moderate, severe) and whether the fall required immediate medical attention. Since pain is a subjective feeling and the threshold for seeking medical attention varies, the actual severity of injuries may have individual fluctuations in this study. Thus, the level of fall injuries that did not require a medical consultation can only be evaluated on the basis of subjective self-reports. The results of the analyses based on the fractures were aligned with the results of the self-reported level of injury, so the results of the fall outcome questionnaire are likely to be reliable. The number of incidents that were reported as “hurt significantly” or required medical attention remained relatively small, which limits the statistical power of this subcategory. However, combining minor fall injuries (“hurt a little”) with more severe outcomes (“hurt significantly”) would not have been a basis upon which to study different levels of fall injury.

## Conclusion

Fall injury prevention should aim to support a physically active lifestyle, maintaining functional capacity, and favorable body composition. Postmenopausal women with higher femoral neck BMD appear to sustain more frequent falls, although they have a lower risk of severe fall injuries, such as fractures. Better TUG and single leg standing tests are protective predictors against falls and fall injuries.
